# Immunophenotyping of Patients With Rheumatoid Arthritis Reveals Difference in CD27^+^IgD^+^ Unswitched Memory B Cell Profiles

**DOI:** 10.1155/mi/9675331

**Published:** 2025-07-29

**Authors:** Bérénice Hansen, Raul Da Costa, Dominique Revets, Fanny Hedin, Maria Konstantinou, Eduardo Rosales Jubal, Franck Ngangom, Cédric C. Laczny, Kirsten Roomp, Viacheslav Petrov, Andreas Michalsen, Etienne Hanslian, Daniela A. Koppold, Anika Rajput Khokhar, Nico Steckhan, Michael Jeitler, Brit Mollenhauer, Sebastian Schade, Michel Vaillant, Antonio Cosma, Paul Wilmes, Jochen G. Schneider

**Affiliations:** ^1^Luxembourg Centre for Systems Biomedicine, University of Luxembourg, Esch-sur-Alzette, Luxembourg; ^2^Department of Translational Medicine Operations Hub (TMOH), Luxembourg Institute of Health, Esch-sur-Alzette, Luxembourg; ^3^Department of Medical Informatics, Luxembourg Institute of Health, Strassen, Luxembourg; ^4^Epidemiology and Health Economics, Institute of Social Medicine, Charité – Universitätsmedizin Berlin Corporate Member of Freie Universität Berlin and Humboldt-Universität zu Berlin, Berlin, Germany; ^5^Department of Internal Medicine and Nature-Based Therapies, Immanuel Hospital Berlin, Berlin, Germany; ^6^Department of Dermatology, Venereology and Allergology, Charité Universitätsmedizin Berlin, Berlin, Germany; ^7^Digital Health-Connected Healthcare, Hasso Plattner Institute, University of Potsdam, Potsdam, Germany; ^8^Institute for General Practice and Interprofessional Care, University Hospital Tuebingen, Tuebingen, Germany; ^9^Robert Bosch Center for Integrative Medicine and Health, Bosch Health Campus, Stuttgart, Germany; ^10^Department of Neurology, University Medical Center Göttingen, Göttingen, Germany; ^11^Paracelsus-Elena-Klinik Kassel, Kassel, Germany; ^12^Department of Life Sciences and Medicine, University of Luxembourg, Esch-sur-Alzette, Luxembourg; ^13^Department of Internal Medicine, Saarland University Hospital and Saarland University Faculty of Medicine, Homburg, Germany

**Keywords:** autoimmunity, CyTOF, IgD^+^CD27^+^ unswitched memory b cells, immunology, immunophenotyping, rheumatoid arthritis

## Abstract

**Objectives:** Over the past decades, the prevalence of noncommunicable diseases has surged significantly, including the systemic autoimmune disorder rheumatoid arthritis (RA). Despite extensive research and advancement of RA therapy, effective prevention strategies or cures remain elusive, and the mechanisms underlying RA pathogenesis unclear. It is crucial to gain deeper insights into RA pathophysiology. The objective of this study is to provide a comprehensive immunophenotyping of patients with RA.

**Methods:** We generated and analyzed deep immunophenotyping data from 52 patients with RA and 47 healthy controls (HCs). Whole blood samples were stained with extracellular markers, and intracellular antibodies and analyzed for 32 different cell markers using mass cytometry by time of flight. The acquired data was analyzed by both manual and automatic unsupervised tools and subsequently complemented with anthropometric data and clinical-laboratory parameters.

**Results:** We observed a significant disparity in immune cell profiles between patients with RA and HC, notably a reduced frequency of CD27^+^IgD^+^ unswitched memory B (_m_B) cells in patients with RA (*p*-value < 0.01), with the disease RA being the primary and only significant factor explaining up to 17.9% of the variance of these cells.

**Conclusion:** Our results reveal, for the first time, that a reduced frequency of unswitched _m_B cells in patients with RA is the only significant abnormality distinguishing patients with RA from HC in a complex immunophenotyping panel of 72 different cell populations. This provides important information to further individualize various interventions and possibly help to design novel therapeutic interventions.

## 1. Introduction

Western societies are burdened by an increased incidence of noncommunicable diseases, resulting in declining overall health and complications later in life [1, 2]. Rheumatoid arthritis (RA) is a non-communicable disease, affecting about 1% of the worldwide population, with women being at a threefold higher risk compared to men [3]. RA is a systemic autoimmune disease, primarily affecting the synovial lining of the joints [4]. The chronic systemic inflammation may also involve the lungs, vasculature, and bones. Clinically, this polyarthritis manifests primarily in joint pain and untreated in mutilating joint destruction, eventually severely impacting the quality of life of patients. The pathogenesis of RA involves a complex interaction of immune cells, including T cells, B cells, dendritic cells, natural killer cells, and monocytes [5, 6]. CD4^+^ T cells have been described as pivotal in RA, interacting extensively with B cells, dendritic cells, and fibroblast-like synoviocytes [5, 6]. B cells contribute to RA by producing autoantibodies such as rheumatoid factor and anti-citrullinated protein antibodies, which are detectable in patients with seropositive RA, and present antigens to T cells [7]. Most mature B cells can be classified into four different subtypes based on the IgD and CD27 surface markers (Table 1). CD27^+^ cells are larger and possess immunoglobulin-producing capabilities [8]. It has been suggested that unswitched _m_B cells are innate-like B cells (ILB) or circulating marginal zone B cells, which develop independently of the germinal center response [9, 10]. Metabolic reprograming leads to the differentiation of human unswitched memory B cells into plasmablasts or CD27^−^IgD^−^ memory B cells [9]. Despite undergoing somatic hypermutation, unswitched _m_B cells do not undergo class switch recombination. A distinctive feature of this subset is their role in the first line of defense. They produce IgM in response to pathogens, which has also been hypothesized to have a protective role in autoimmune diseases [9, 10]. RA is a highly heterogenous disease, and the immunophenotype goes far beyond the simple classification of seropositive or seronegative RA [11]. Patients have been reported to respond differently to various treatment options, show different progression rates, distinct comorbidities, and overall differing phenotypes [12]. Likely, the clinical features depend on the heterogenous immunophenotype in a nonlinear fashion. Furthermore, several cell subsets are known to respond in an environment-dependent way, including dendritic cells, *γ*δ T cells, B cells, and natural killer cells [13, 14]. To date, the disease is treated with anti-inflammatory drugs for the acute flares and with long-acting immune suppressing or modulating therapies to influence the course of the disease. Both a sustainable and efficient prevention as well as a cure are currently lacking [15]. Accumulating evidence suggests a pivotal role for environmental factors, including nutrition, physical activity, lifestyle interventions like caloric restriction, and the gut microbiome, in RA pathophysiology [16]. Yet, elucidating the immunophenotypic landscape of this chronic inflammatory disorder and the immunological pathways influenced by therapeutic interventions remain key for advancing our understanding of RA pathogenesis and treatment strategies.

To deeper understand the immunophenotypic landscape of RA, we characterized the blood of patients with RA using CyTOF, employing a high-dimensional approach to detect marker combinations and cluster generation taking into consideration clinical and laboratory features [4]. We aimed to compare the obtained profile to healthy controls (HCs), displaying distinct immunological differences to enable better understanding of pathophysiology and open opportunities for individual, targeted treatments [17].

## 2. Results

### 2.1. Clinical Cohorts

A final number of 99 samples was included in the analysis, including 47 HC and 52 patients with RA. We omitted 21 samples due to missing values, either immunological or clinical, resulting in *n* = 52 patients with RA and *n* = 47 HCs. The clinical and anthropometrical characteristics are summarized in Table 2. Overall, the clinical disease activity score (CDAI) was high for the patients with RA, signaling a state of active disease and acute flares [18] (Table 3). We observed statistically significant differences between the two cohorts: for patients with RA, we observed a lower number of hours of sleep, lower waist–hip ratio (WHR), reduced frequency of walking for longer than 10 min and lower creatinine levels compared to HC.

### 2.2. High-Dimensional Comparison of Immune Cell Profiling in Patients With RA and HC

**Figure 1:**
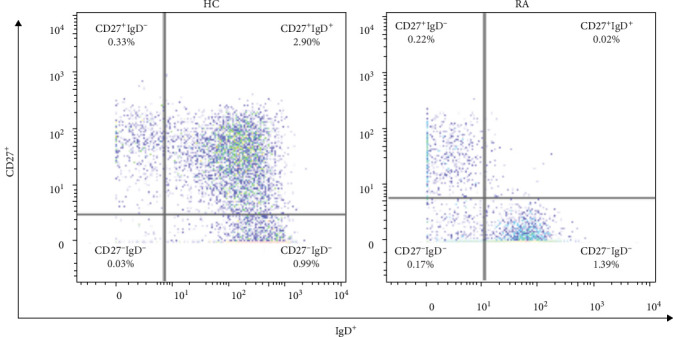
Representative CD27 IgD dot plots from a healthy control (HC) and a patient with rheumatoid arthritis (RA) indicating the distribution and percentages of the following memory B cell subsets in the total CD45^+^ cell population: CD27^+^IgD^+^ (unswitched); CD27^+^IgD^−^ (switched); CD27^−^IgD^+^ (naïve); CD27^−^IgD^−^ (double negative).

**Figure 2:**
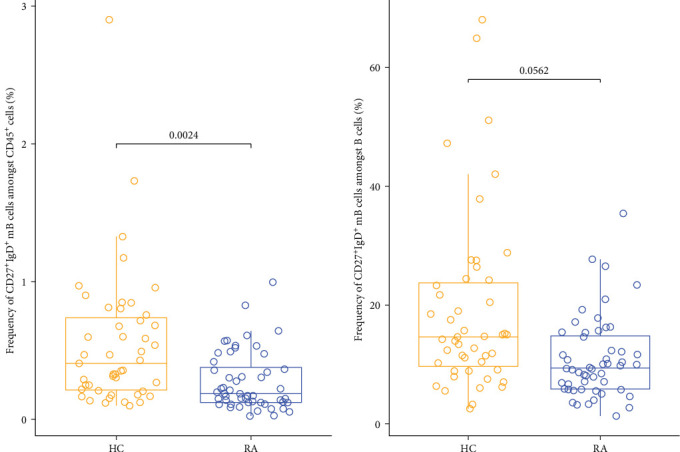
Differences in cell expression in unswitched memory B (_m_B) cells in patients with rheumatoid arthritis (RA) compared to healthy controls (HCs). The left plot illustrates the frequency of unswitched _m_B cells as a proportion of the overall population of CD45^+^ cells, whereas the right plot depicts the frequency of unswitched _m_B cells relative to the total B cell population.

We compared immune cell frequencies between patients with RA and HCs. Most cell types studied did not exhibit significant differences after correcting for multiple comparisons. However, we found significantly lower cell frequencies of unswitched _m_B cells in patients with RA compared to HC in the supervised, hierarchical analysis in both total CD45^+^ cells (*p*-value = 0.0032) and total B cells (*p*-value = 0.0098; Figures 1 and 2). Although the *γ*δ T cells also trended to be lower in patients with RA (Figure S5), this difference did not reach statistical significance after adjustment for multiple comparisons (*p*-value > 0.05 in FDR) [19, 20]. For the automated unsupervised analysis performed by the CellEngine software, the best distribution and differentiation of the cells displayed by the expression of their respective markers in the form of a heatmap could be observed with a total of 100 clusters (10 × 10). To avoid dispersion of neutrophil populations into numerous clusters, and thus conceal less frequent cell subsets, a more targeted approach was applied to better define the cluster identities. This was done by applying the 10 × 10 clustering to a cell subset, excluding neutrophils (Figure S6). No significant differences were observed between the two groups in the unsupervised analysis. Together, the supervised immunophenotyping demonstrated a significantly lower number of unswitched _m_B cells in patients with RA compared to HC.

### 2.3. Integration of Clinical Data and Treatment

**Figure 3:**
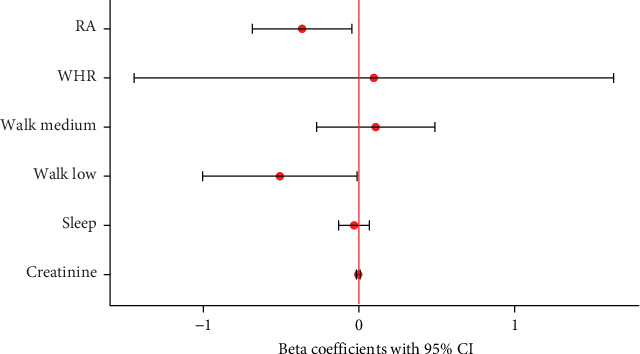
Impact of different factors on unswitched memory B (_m_B) cell frequency in patients with rheumatoid arthritis (RA) and healthy controls (HCs). RA is the primary and only significant factor explaining up to 17.9% of the variance of unswitched mB cells in this model. WHR, waist–hip ratio; Walk, medium and walk, low; a medium and low frequency of walking for longer than 10 min; sleep, hours of sleep; Creatinine, creatinine levels µmol/L.

**Figure 4:**
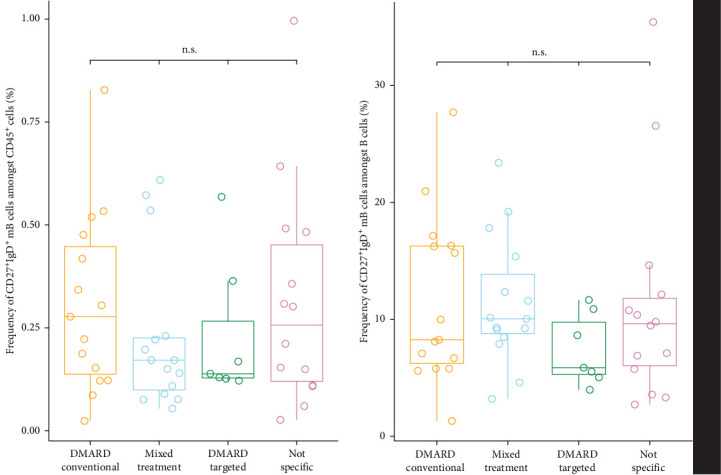
Impact of different treatments on cell expression in unswitched memory B (_m_B) cells in patients with rheumatoid arthritis (RA) compared to healthy controls (HCs). The left plot illustrates the frequency of unswitched _m_B cells as a proportion of the total B cell population, whereas the right plot depicts the frequency of unswitched _m_B cells relative to the overall population of CD45^+^ cells, based on the hierarchical analysis. The patients were separated into four groups, based on their different treatments. No significant differences between the treatment groups were observed.

Potential confounding factors did not significantly correlate, confirming their suitability for our models S7. Two linear regression models were constructed to assess the impact of various factors on unswitched _m_B cell frequencies in patients with RA and HC. The first model, including both RA patients and HC, revealed that RA was the only significant predictor of unswitched _m_B cell frequency (CD45^+^ parent population: β = −0.752, *p*  < 0.01; B cell parent population: β = −0.365, *p*  < 0.05), independent of other covariates (WHR, creatinine, sleep). Only walking showed a moderate impact (*p*=0.043). This model explained 17.9% of the variance in unswitched _m_B cell levels in the CD45^+^ cell population and 16.9% in the total B cell population (*p*  < 0.01) (Figure 3). The second linear regression model, focusing on potential confounders within the RA group alone, indicated that none of the variables, including disease duration and medication type, significantly impacted unswitched _m_B cell frequency (Figure S8). This model also exhibited a low adjusted *R*-squared value, suggesting that the included confounders did not contribute substantially to explain the variation in cell measurements. We also corrected for the impact of medication on the unswitched _m_B cell frequency. We differentiated between five different treatment groups: conventional DMARDs, biologic and targeted synthetic DMARDs, glucocorticoids, combined treatment, and no specific RA medication (Figure S9). The conventional DMARDs consisted mostly of methotrexate, but also included Leflunomide and Sulfasalazine. The treatments in the biologic and targeted DMARD group were Adalimumab, Sarilumab, Golimumab, Etanercept, Tocilizumab, and Baricitinib. The glucocorticoid medication was exclusively Prednisolone. We detected no significant difference in unswitched _m_B cells between the different medication treatment groups (Kruskal–Wallis test by ranks) [21] (Figure 4). Hence, the significantly lower number of unswitched _m_B cells in patients with RA was not impacted by any confounder or medication and might be a key characteristic of the autoimmune disease.

## 3. Discussion

Regarding the phenotypic characterization, we found several differences, namely creatinine, duration of sleep, walking frequency, and WHR, in clinical and anthropometrical characteristics between patients with RA and HCs, some of which might be explained by the nature of the disease [22–24]. The slightly reduced amount of sleep recorded for patients with RA might be explained by typical RA symptoms such as joint pain and stiffness, as well as side effects of medication or comorbidities, including anxiety and depression and a higher prevalence of sleep-related breathing disorders, leading to sleep disturbance [25]. Also, although walking is classified as a feasible, safe, and beneficial intervention for patients with RA, these patients spent generally less time walking than the HCs, which might be associated with joint pain and other RA symptoms like fatigue [24]. Creatinine levels were significantly lower in patients with RA, which could be due to an increased risk for sarcopenia in RA or side effects of RA specific medication [26, 27]. The observed lower WHR in patients with RA is atypical as the chronic inflammation in combination with medication and reduced physical activity often leads to an increased WHR compared to HC [28]. We expected to observe a distinct immunological profile in patients with RA compared to controls and were able to confirm this in our analysis. Despite the ongoing medical treatment, we found a significantly lower number of unswitched _m_B cells in patients with RA compared to HCs. Additionally, *γ*δ T cells were lower in patients with RA (Figure S9). However, after correction for multiple testing the difference was no longer significant. Previous studies have reported elevated B cell numbers in RA, which is a phenomenon generally anticipated in autoimmune diseases [29]. Treatment strategies therefore often focus on B cell depletion in such scenarios due to the pivotal role of these cells in antigen presentation, cytokine, and antibody production [30]. Conversely, for the specific unswitched _m_B cell subset, lower levels have been previously reported for patients with systemic lupus erythematosus (SLE), patients with Sjogren's syndrome as well as for patients with RA compared to control subjects [9, 31]. Although unswitched _m_B cells carry higher potentials of inflammatory response than CD27^−^ B cells, they have been reported to negatively correlate with disease activity [10].

Also, it was found that in B-cell depletion therapy-naïve patients with RA, the frequency of unswitched _m_B cells correlated inversely with levels of serum B cell activation [32]. In addition, differences in serum immunophenotypes of B cells in autoantibody-positive and -negative RA have been found and add to the complexity of the immunophenotyping of patients with RA [33]. Their function was found to be impaired, including a decreased IgM production [10]. As IgM antibodies have been recognized to play protective roles in autoimmune diseases, one possible key role of unswitched _m_B cells in RA could be linked to reduced IgM producing capacities [10]. Another aspect of B cells is their B cell receptor repertoire (BCR). Recent studies have reported abnormalities for autoimmune diseases such as RA and SLE. In SLE, VDJ gene usage is notably skewed, especially in unswitched _m_B cells and plasmablasts, indicating a potential important role of the latter in RA as well [34]. Also, the possible migration of the cells to the inflamed synovium or the differentiation into other subsets might explain the reduced levels of these unswitched _m_B cells found in patients with RA [9]. Although the exact role of the unswitched _m_B cell subset in RA is not clear yet, a major role is suggested, with the disease RA being the primary and only significant factor explaining up to 17.9% of the variance of unswitched _m_B cells in our model. The previously reported findings and our results emphasize the importance of better understanding the role of unswitched _m_B in autoimmunity and optimizing treatment and prevention in patients with RA. *γ*δ T cells have also been previously reported to be lower in patients with RA in 1999 [35]. This finding has then been both confirmed and challenged in the past years and could not be confirmed in this study [35, 36]. Several cell subsets have been reported to adapt their response according to the environment to either pro-inflammatory or tolerogenic response [37]. This is also the case for specific subsets of *γ*δ T cells that have been suggested to play an important role in inflammatory response [36]. *γ*δ T cells can exert different effector phenotypes, amongst others cytotoxicity, cytokine production, and immunoregulatory functions [13, 38]. This environment-dependent response of some immune cell subsets is a crucial aspect that must be considered for future research, prevention, and treatment therapies. Both the heterogeneity of RA and the adaptive cell-response might explain why, besides the difference in unswitched _m_B cells, no obviously distinct pattern could be detected comparing patients with RA to HCs. Although reduced numbers of unswitched _m_B cells in patients with RA have been reported in 2009 and 2018 by using specific staining (e.g., CD19, CD27, and IgD) on isolated PBMCs or whole blood by using FACS or flow cytometry, respectively, we show for the first time that a reduced frequency of unswitched _m_B cells is the only significant difference distinguishing the immunophenotype from patients with RA from HC in a complex immunophenotyping panel of 72 different cell populations [10, 31].

The findings of our study highlight the complex and dynamic role of the immune system in the chronic, systemic, autoimmune disease RA. Understanding the underlying mechanisms and consequences of the reduced number of unswitched _m_B cells in patients with RA could provide valuable insights into RA pathogenesis and lead to the development of more targeted and effective therapeutic strategies. Further research is necessary to elucidate the precise role and underlying mechanisms of these cells and their potential as biomarkers or therapeutic targets in RA. Investigations focusing on longitudinal studies with targeted interventions, could help clarify the role of unswitched memory B cells, particularly in relation to CDAI fluctuations.

## 4. Methods

### 4.1. Sample Collection

The samples for the CyTOF analysis were collected as part of the ExpoBiome study [4]. The patients were either diagnosed with RA or classified as HCs and included according to the exclusion and inclusion criteria [4]. HCs were without any evidence of active known or treated RA. The cohorts were matched for age and gender. Ethical approval was given by the Ethics Committee of Charité-Universitätsmedizin Berlin (EA1/204/19), the Ethics Committee of the State Medical Association of Hesse (2021-2230-zvBO) and the Ethics Review Panel (ERP) of the University of Luxembourg (ERP 21-001A ExpoBiome). The study was registered in Clinicaltrials (https://clinicaltrials.gov/ct2/show/NCT04847011).

### 4.2. Sample Processing and Extracellular Staining for CyTOF

Immediately after blood collection in heparin tubes, the whole blood samples were stained with the MaxPar Direct Immune Profiling Assay (MDIPA, Standard Biotools, CA, USA) and stabilized with Prot1 stabilizer (SmartTube Inc., San Carlos, CA, USA) according to a previously validated workflow [39] Supporting Information Figure S1. The samples were stored at −80°C until further processing.

### 4.3. Intracellular Staining and Acquisition for CyTOF

Before additional intracellular staining with the in-house conjugated antibodies, all antibodies have been titrated (Figure S2). The subsequent sample preparation was done according to the manufacturer's protocol (Standard Biotools, CA, USA). The whole blood samples were thawed in a 12°C water bath before a thaw lyse buffer, prepared from a 1:2000 dilution of 1000x concentrate (SmartTube Inc., San Carlos, CA, USA) and MilliQ water was added. The samples were incubated for 10 min. After centrifugation, MaxPar Cell Staining Buffer (CSB, 201068, Standard Biotools, CA, USA) was added to the sample. The samples were treated with the eBioscience Foxp3/Transcription Factor Staining Buffer Set (00-5523-00, Invitrogen, MA, USA) and then stained with the optimal concentration of in-house conjugated antibodies before a 30 min incubation at 4°C. After several washing steps, a freshly prepared 1.6% formaldehyde solution (Pierce, 16% Formaldehyde, 289006, Thermo Fisher Scientific) was added to the samples. A multiplexing strategy based on the use of the Cell-ID 20-Plex Pd Barcoding Kit (Cell-ID, 201060, Standard BioTools, CA, USA) was applied, which enabled the analysis of up to 20 samples per experiment (Figure S1). A DNA Ir-intercalator solution (201192A, Standard Biotools, CA, USA) was prepared. After dissolving the barcodes in 100 µL of a diluted Ir-solution and adding them to the samples, the cells were resuspended in the remaining DNA Ir-intercalator solution to a final concentration of 50 nM Ir-Intercalator per 3 × 10^6^ cells and incubated overnight at 4°C. Prior to the acquisition, the barcoded cells were pooled together and underwent further washing steps with Maxpar Cell Acquisition PLUS (CAS PLUS, 201244, Standard Biotools, CA, USA). 10% calibration beads (Maxpar Four Elements EQ Beads, 201078, Standard Biotools, CA, USA) were added to the sample before the acquisition with a Helios mass cytometer (Standard BioTools, CA, USA). To avoid batch effects and monitor technical variation, the sample acquisition was randomized, and a reference sample was included in each CyTOF run (Figure S2).

### 4.4. Hierarchical Gating and Unsupervised Analysis of CyTOF Data

After the CyTOF acquisition, the Flow Cytometry Standard (FCS) data files were debarcoded using an integrated debarcoder tool in the CyTOF software 7.1 according to the manually set Minimum Barcode Separation parameter. The newly generated debarcoded FCS files were imported into FlowJo v10.9 Software (BD Life Sciences, Ashland, USA), and a gating strategy was established to identify and characterize 72 relevant immune cell populations (Figure S3). Information on the number of each cell population and their respective parental frequencies (%) was exported for further analysis of the hierarchical and supervised data. Using Tableau Prep Builder v2021.4 (Tableau Software, LLC, Washington, USA), a pipeline to prepare and organize the exported data was generated [40]. Additional sample information was added to the pipeline, and the consequent database used in Tableau Desktop v2023.2 (Tableau Software, LLC, Washington, USA) included additional metrics.

In addition to the hierarchical gating analysis, we performed an automated, unsupervised analysis clustering cell populations based on similar protein marker expression profiles [41]. This analysis was performed using the CellEngine software (CellCarta, Montreal, Canada). Preliminary tests using FCS with data on CD45^+^ cells were run to evaluate different FlowSOM parameters, notably, the numbers of final clusters and the number of consensus clusters. We tested different conditions, including 10 × 10 and 12 × 12 final clusters with 12 or 24 consensus clusters. The expression of the different markers visualized as heatmaps was used to identify the cell populations representing the different clusters in an unbiased way. After establishing the identity of the major cell populations with the newly generated heat map, the cluster ID information was imported into the Tableau Prep pipeline.

### 4.5. Statistical Analysis of Hierarchical and Unsupervised Analysis of CyTOF Data

The calculated metrics and resulting data were imported into Qlucore Omics Explorer 3.8.1 (Qlucore, Sweden) to assess statistical differences between clusters or cell populations and cohorts by calculating *p*-values and *Q*-values, using different parent populations as reference for the hierarchical dataset (CD45^+^ Live, B-cells, CD3^−^, CD19^−^, CD4^+^, CD8^+^, MAIT). The Mann–Whitney *U* test was applied to the hierarchical and unsupervised dataset.

### 4.6. Clinical Data Integration and Statistical Analysis

Clinical data collected in REDCap was integrated into the data acquired by CyTOF using R (R 4.3.2) and R studio (2023.09.1 + 494) (Posit PBC, Boston, USA). The baseline characteristics of patients with RA and HC have been compared using a Mann–Whitney *U*-test. Possible confounders were selected, including age, gender, diet, WHR, body mass index (BMI), wellbeing score (WHO-5), hours of sleep and time spent walking, albumin, creatinine, insulin, C-reactive protein (CRP), cholesterol, glucose, and thyroid stimulating hormone (TSH) levels. The selected integrated factors were tested for correlation to ensure they were independent. To test these possible confounding factors, a linear regression model with the disease RA as predictor was built. Variables were visually inspected using histograms, tested for normality by applying the Shapiro–Wilk-test, and log-transformed when necessary to meet linear regression assumptions. A second linear regression model was built to define further explanatory factors for acquired cell counts in patients with RA. This model looked at specific RA predictors including the duration of disease, CDAI, rheumatoid factor, and CRP. The *p*-values were adjusted according to the Benjamini–Hochberg false-discovery rate method for each model to correct for multiple testing [19, 20]. A *p*-value < 0.05 was regarded as statistically significant. In addition, as most of the patients with RA were not treatment-naïve at the time of the blood sampling, a Kruskal Wallis test was run to account for different treatment groups (Figure S4) [21].

### 4.7. Limitations of the Study

The limitations of the study include a reduced sample size, as the sample size of initially *n* = 120 patients was reduced to *n* = 99 patients due to missing clinical values. The analysis in patients with RA was done on *n* = 52 patients, which is further limited by different medical treatments of the patients. As the study was designed to compare the immunophenotype of patients with RA to HCs, this study lacks the statistical power (power = 0.13) to deeply analyze the effects of medication, heterogenous RA pathophysiology, and environment-dependent cell response. However, this analysis was not the aim of this study and should be further investigated in future clinical trials.

## Figures and Tables

**Table 1:** Memory B cell subtypes based on their cell surface expression of markers CD27 and IgD.

Memory B cell subtype	CD27	IgD
Unswitched	+	+
Switched	+	−
Naïve	−	+
Double negative	−	−

**Table 2:** Baseline characteristics of patients with RA (*n* = 52) and healthy controls (*n* = 47).

	Rheumatoid arthritis (*n* = 52)		Control group (*n* = 47)		*p* value
Variable	Median	IQR	Median	IQR	
Age (y)	55.23	12.56	57.23	14.93	0.5656
Female (%)	88.3	—	72.1	—	0.0648
BMI	23.94	5.83	24.21	5.76	0.5752
WHR*⁣*^*∗∗*^	0.81	0.07	0.88	0.1	0.0004
WHO-5	13.5	8	17	9.5	0.0569
Sleep (h)*⁣*^*∗*^	6	2	7	2	0.0118
Walking for 10 min*⁣*^*∗∗*^	2	1	2	0	0.0055
Diet (% omnivore)	53.1	—	61	—	0.3029
Albumin (g^*⁣*^*∗*^^L^−1^)	42.5	4.075	43.1	2.6	0.0679
Creatinine (µmol*⁣*^*∗*^L^−1^)*⁣*^*∗∗*^	58.5	11.88	66.7	11.85	0.0000
hs-CRP (mg*⁣*^*∗*^L^−1^)	1.7	2.55	1.04	1.085	0.6948
Insulin (mU/L^−1^)	6.4	3.45	5.5	3.1	0.1750
TSH basal (mU*⁣*^*∗*^L^−1^)	0.99	0.68	1.11	0.85	0.1997
Cholesterol (mmol*⁣*^*∗*^L^−1^)	5.83	1.35	5.59	1.745	0.7792
Glucose (mmol*⁣*^*∗*^L^−1^)	5.035	0.7525	4.98	1.0775	0.6513

Abbreviations: BMI, body mass index; CRP, C-reactive protein; WHO-5, wellbeing score; WHR, waist–hip ratio; y, years.

*⁣* ^*∗*^ *p*-value < 0.05.

*⁣* ^*∗∗*^ *p*-value < 0.01 (RA vs HC).

**Table 3:** Specific baseline characteristics of patients with RA (*n* = 52).

Variable	Median	IQR
CDAI	55	82.75
Rheumatoid factor (IU mL^−1^)	18.3	37.3
Disease duration (y)	7.25	13.76

Abbreviations: CDAI, clinical disease activity index; y, years.

## Data Availability

All relevant patient data used in this study can be requested by contacting the corresponding authors.

## Supporting Information

Supporting Information Table S1: MDIPA extracellular antibodies used to stain whole blood samples. Table S2: Antibodies for intracellular staining of the extracellular stained and stabilized whole blood samples for CyTOF anaylsis, coupled in-house. Figure S1: Representation of the barcoding patterns. Figure S2: tSNE analysis of the reference sample pooled together. Figure S3: FlowJo Gating strategy. Figure S4: Flowchart of statistical analysis. Figure S5: Distribution of *γ*δ T cells. Figure S6: Unsupervised clustering analysis. Figure S7: Spearman correlation for selected covariates. Figure S8: Impact of different factors on unswitched memory B (mB) cell frequency in patients with rheumatoid arthritis (RA) and healthy controls (HCs). Figure S9: Different treatment groups.
