# Supplementary material for: Immunophenotyping of Patients With Rheumatoid Arthritis Reveals Difference in CD27+IgD+ Unswitched Memory B Cell Profiles
Source: Mediators Inflamm. 2025 Jul 29;2025:9675331. doi: 10.1155/mi/9675331 (PMC12324914; doi:10.1155/mi/9675331)
Supplement: Supporting Information — Table S1: MDIPA extracellular antibodies used to stain whole blood samples. Table S2: Antibodies for intracellular staining of the extracellular stained and stabilized whole blood samples for CyTOF anaylsis, coupled in-house. Figure S1: Representation of the barcoding patterns. Figure S2: tSNE analysis of the reference sample pooled together. Figure S3: FlowJo Gating strategy. Figure S4: Flowchart of statistical analysis. Figure S5: Distribution of γδ T cells. Figure S6: Unsupervised clustering analysis. Figure S7: Spearman correlation for selected covariates. Figure S8: Impact of different factors on unswitched memory B (mB) cell frequency in patients with rheumatoid arthritis (RA) and healthy controls (HCs). Figure S9: Different treatment groups. [file 9675331.f1.docx]

SUPPLEMENTARY TABLES AND FIGURES

Supplementary table 1: MDIPA extracellular antibodies used to stain whole blood samples

| Metal Isotope | Antibody | Clone | Manufacturer | Catalogue# |
| --- | --- | --- | --- | --- |
| 89Y | CD45 | HI30 | Standard Biotools, | Part of MDIPA |
| 103Rh | Live/Dead indicator |  | Standard Biotools | Part of MDIPA |
| 141Pr | CD196 (CCR6) | G034E3 | Standard Biotools | Part of MDIPA |
| 143Nd | CD123 | 6H6 | Standard Biotools | Part of MDIPA |
| 144Nd | CD19 | HIB19 | Standard Biotools | Part of MDIPA |
| 145Nd | CD4 | RPA-T4 | Standard Biotools | Part of MDIPA |
| 146Nd | CD8a | RPA-T8 | Standard Biotools | Part of MDIPA |
| 147Sm | CD11c | Bu15 | Standard Biotools | Part of MDIPA |
| 148Nd | CD16 | 3G8 | Standard Biotools | Part of MDIPA |
| 149Sm | CD45RO | UCHL1 | Standard Biotools | Part of MDIPA |
| 150Nd | CD45RA | HI100 | Standard Biotools | Part of MDIPA |
| 151Eu | CD161 | HP-3G10 | Standard Biotools | Part of MDIPA |
| 152Sm | CD194 (CCR4) | L291H4 | Standard Biotools | Part of MDIPA |
| 153Eu | CD25 | BC96 | Standard Biotools | Part of MDIPA |
| 154Sm | CD27 | O323 | Standard Biotools | Part of MDIPA |
| 155Gd | CD57 | HCD57 | Standard Biotools | Part of MDIPA |
| 156Gd | CD183 (CXCR3) | G025H7 | Standard Biotools | Part of MDIPA |
| 158Gd | CD185 (CXCR5) | J252D4 | Standard Biotools | Part of MDIPA |
| 160Gd | CD28 | CD28.2 | Standard Biotools | Part of MDIPA |
| 161Dy | CD38 | HB-7 | Standard Biotools | Part of MDIPA |
| 163Dy | CD56 (NCAM) | NCAM16.2 | Standard Biotools | Part of MDIPA |
| 164Dy | TCRgd | B1 | Standard Biotools | Part of MDIPA |
| 166Er | CD294 | BM16 | Standard Biotools | Part of MDIPA |
| 167Er | CD197 (CCR7) | G043H7 | Standard Biotools | Part of MDIPA |
| 168Er | CD14 | 63D3 | Standard Biotools | Part of MDIPA |
| 170Er | CD3 | UCHT1 | Standard Biotools | Part of MDIPA |
| 171Yb | CD20 | 2H7 | Standard Biotools | Part of MDIPA |
| 172Yb | CD66b | G10F5 | Standard Biotools | Part of MDIPA |
| 173Yb | HLA-DR | LN3 | Standard Biotools | Part of MDIPA |
| 174Yb | IgD | IA6-2 | Standard Biotools | Part of MDIPA |
| 176Yb | CD127 | A019D5 | Standard Biotools | Part of MDIPA |

Supplementary table 2: Antibodies for intracellular staining of the extracellular stained and stabilized whole blood samples for CyTOF anaylsis, coupled in-house

| Metal Isotope | Antibody | Clone | Manufacturer | Catalogue# | uL/sample |
| --- | --- | --- | --- | --- | --- |
| 116Cd | Bcl-6 | REA373 | Miltenyi | 130-124-533 | 2 |
| 175Lu | IRF4 | IRF4.3E4 | Biolegend | 646402 | 2 |


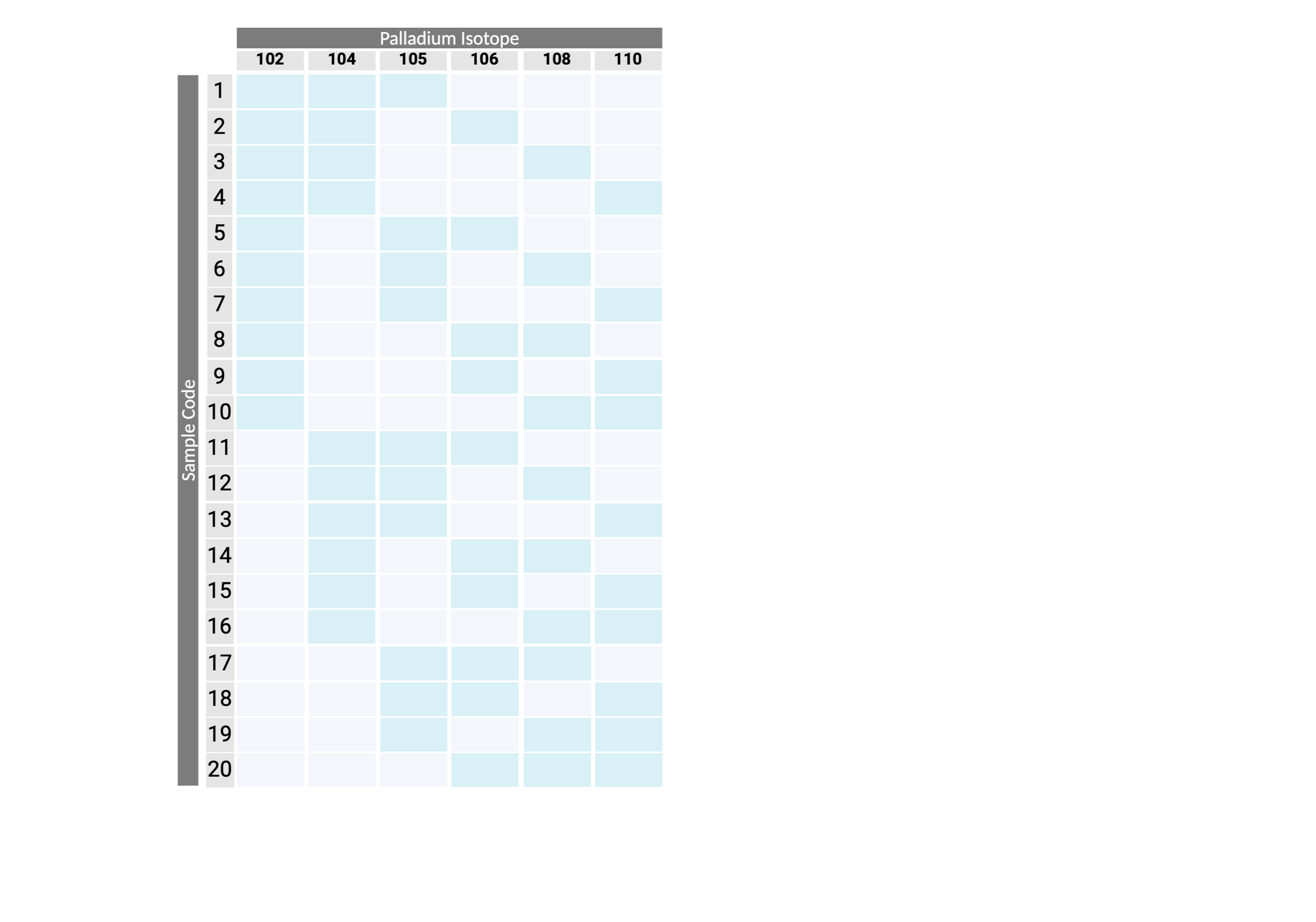


Supplementary figure 1: Representation of the barcoding patterns, each sample code consisting of a unique combination of 3 palladium isotopes. The barcoding allowed the simultaneous acquisition of several samples. Created in Biorender.com

Supplementary figure 2a: tSNE analysis of the reference sample pooled together to the experimental samples prior to each acquisition. After debarcoding the reference sample FCS were analysed by t-SNE and FlowSOM using all cellular surface markers. FlowSOM 100 clusters are shown by a blue to red color scale. Acquisition date is indicated at the top of each graph.

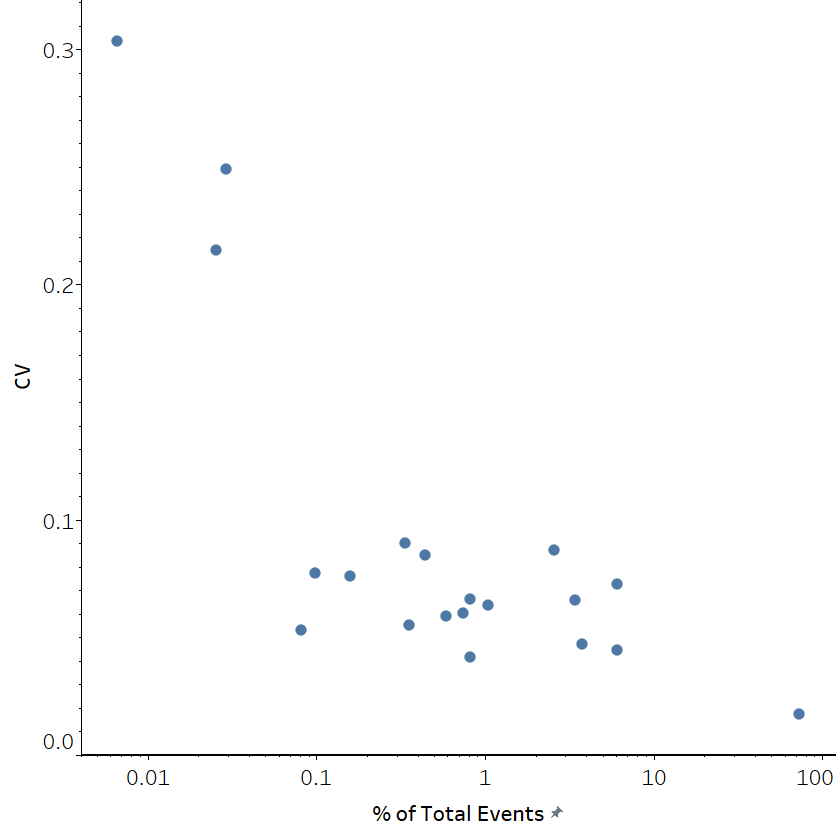


Supplementary figure 2b-d: Supplementary Figure 2b shows the % of each FlowSOM meta-cluster over the acquisition date and Supplementary Figure 2c shows the variation associated to each meta-cluster. We observed a CV<10% in 17 out of 20 meta-clusters (Supplementary Figure 2d). The three meta-clusters with a CV higher than 20% had a low average percentage of cells (<0.04 %).


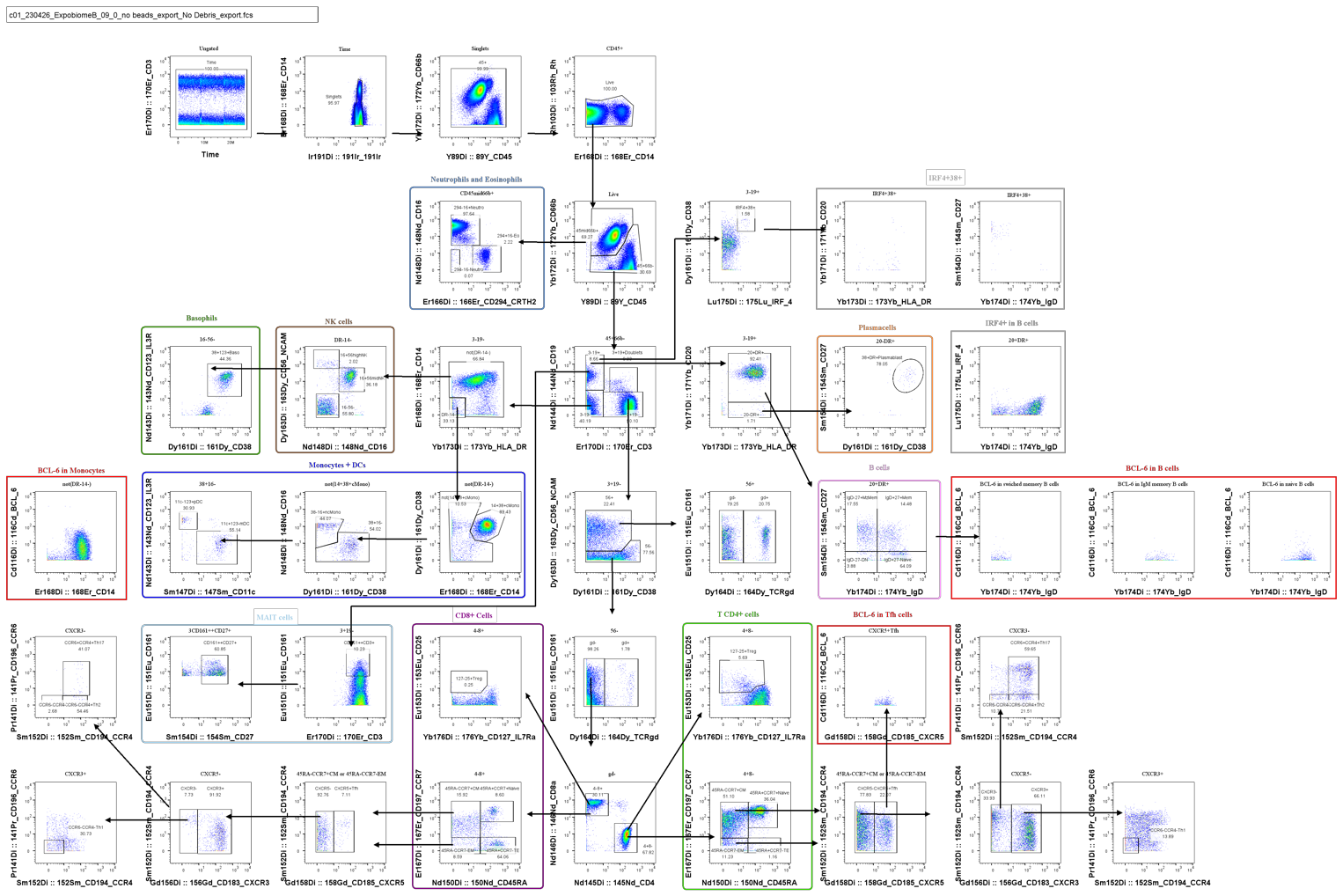


Supplementary figure 3: FlowJo Gating strategy to identify and characterize 72 relevant immune cell populations. The supervised, manual analysis of the acquired data was done based on this gating strategy, which was developed specifically for the data in question.


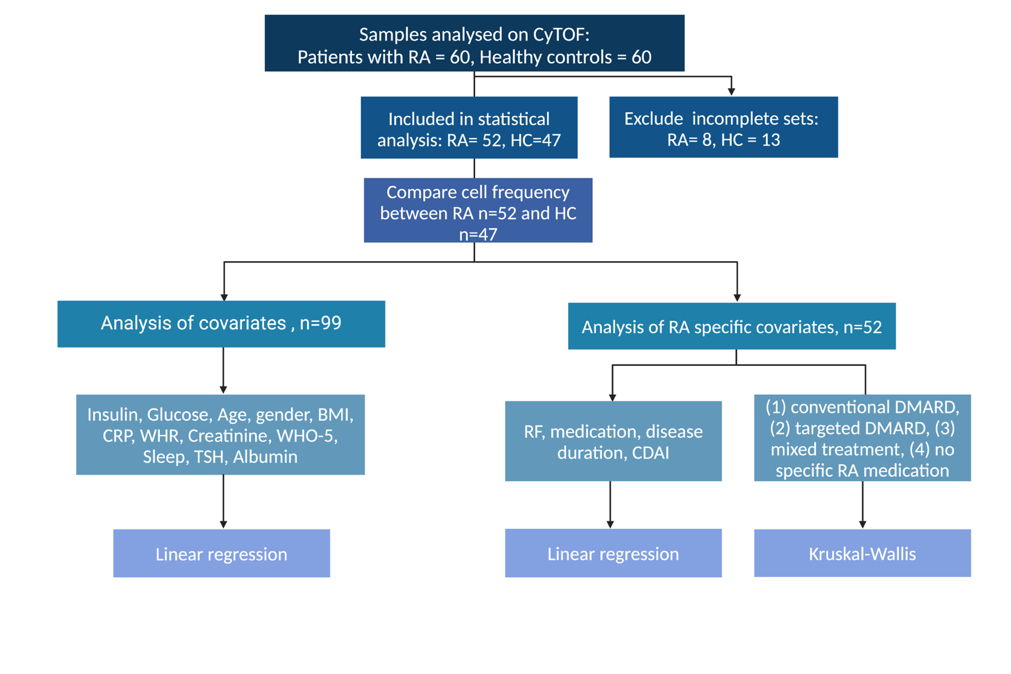


Supplementary figure 4: Flowchart of statistical analysis, showing the different steps of the statistical analysis that has been done including the final number of participants that were considered for the analysis.

*
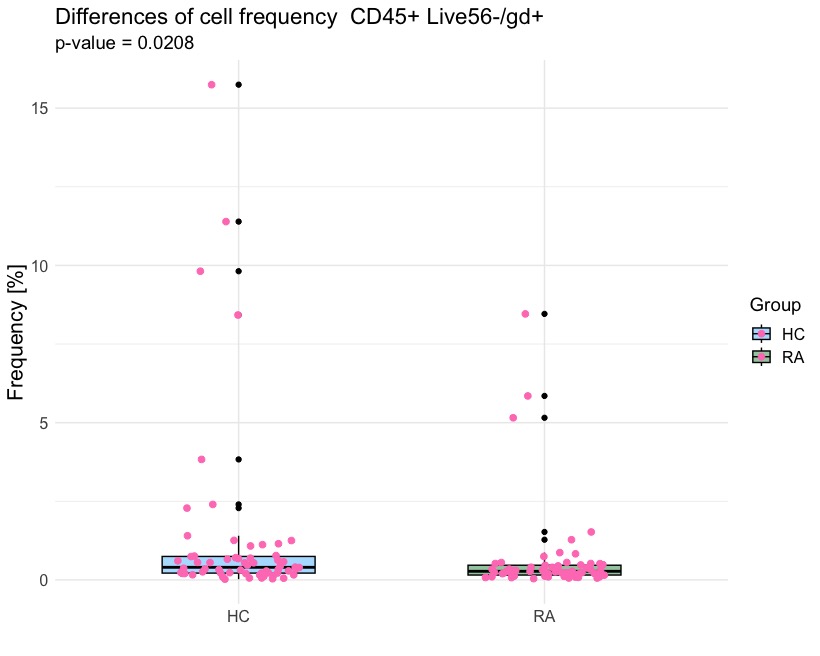
*

Supplementary figure 5: Boxplot representing the *𝛾𝛿* T cell frequency in healthy controls (HC) and patients with rheumatoid arthritis (RA).


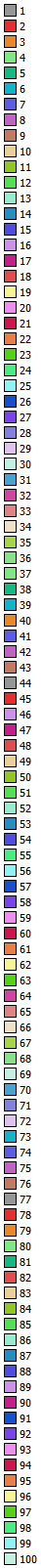


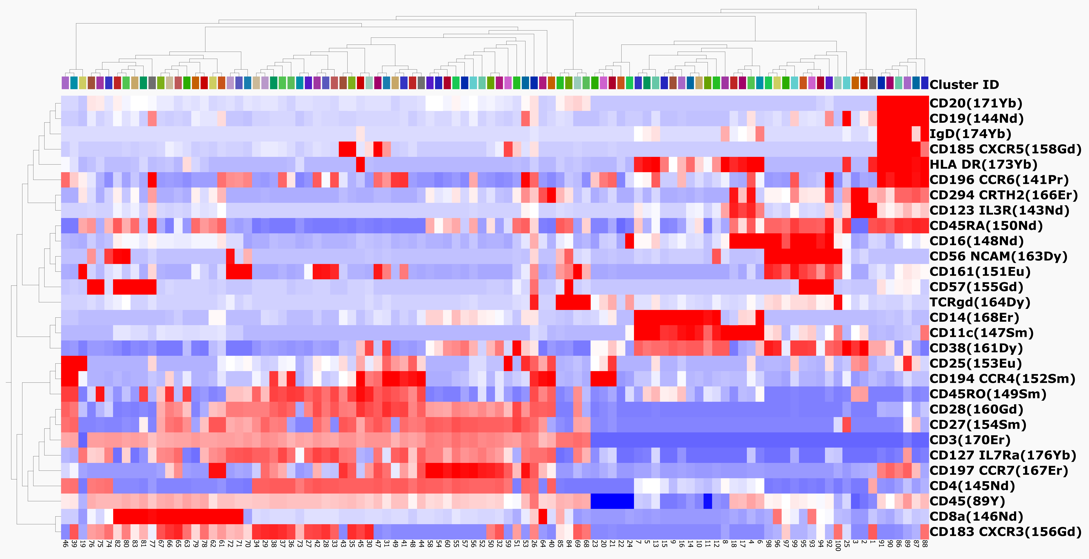

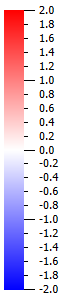


Supplementary figure 6: Unsupervised clustering analysis of differentially expressed cell markers in patients with RA and healthy controls. 10x10 cluster analysis generated by CellEngine, represented as heatmap. Color grade from red (high expression) to blue (low expression). The cell markers are listed on the right.


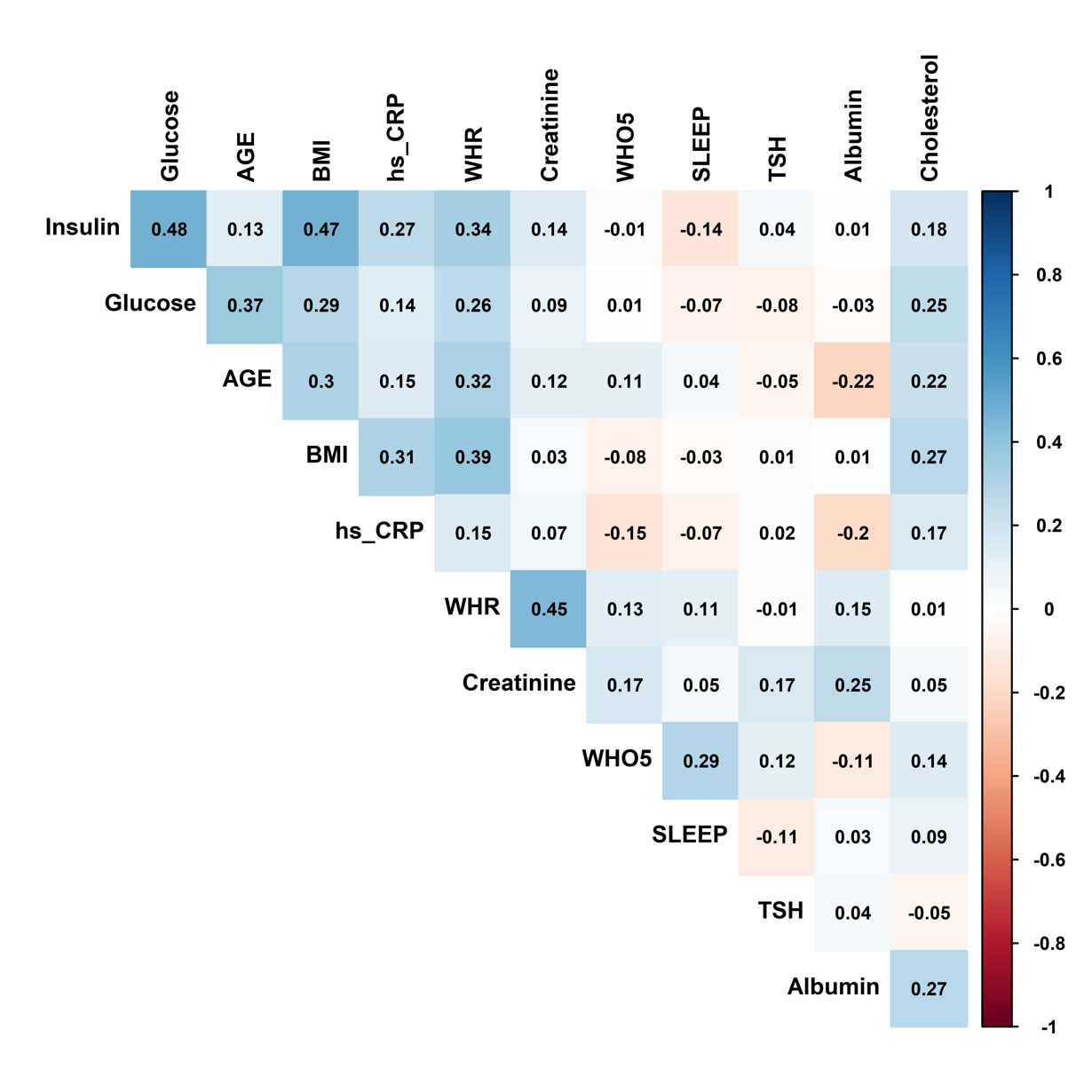


Supplementary figure 7: Spearman correlation for selected covariates to ensure independence of possible confounding factors. The color gradient goes from dark red (strong negative correlation) to dark blue (strong positive correlation).


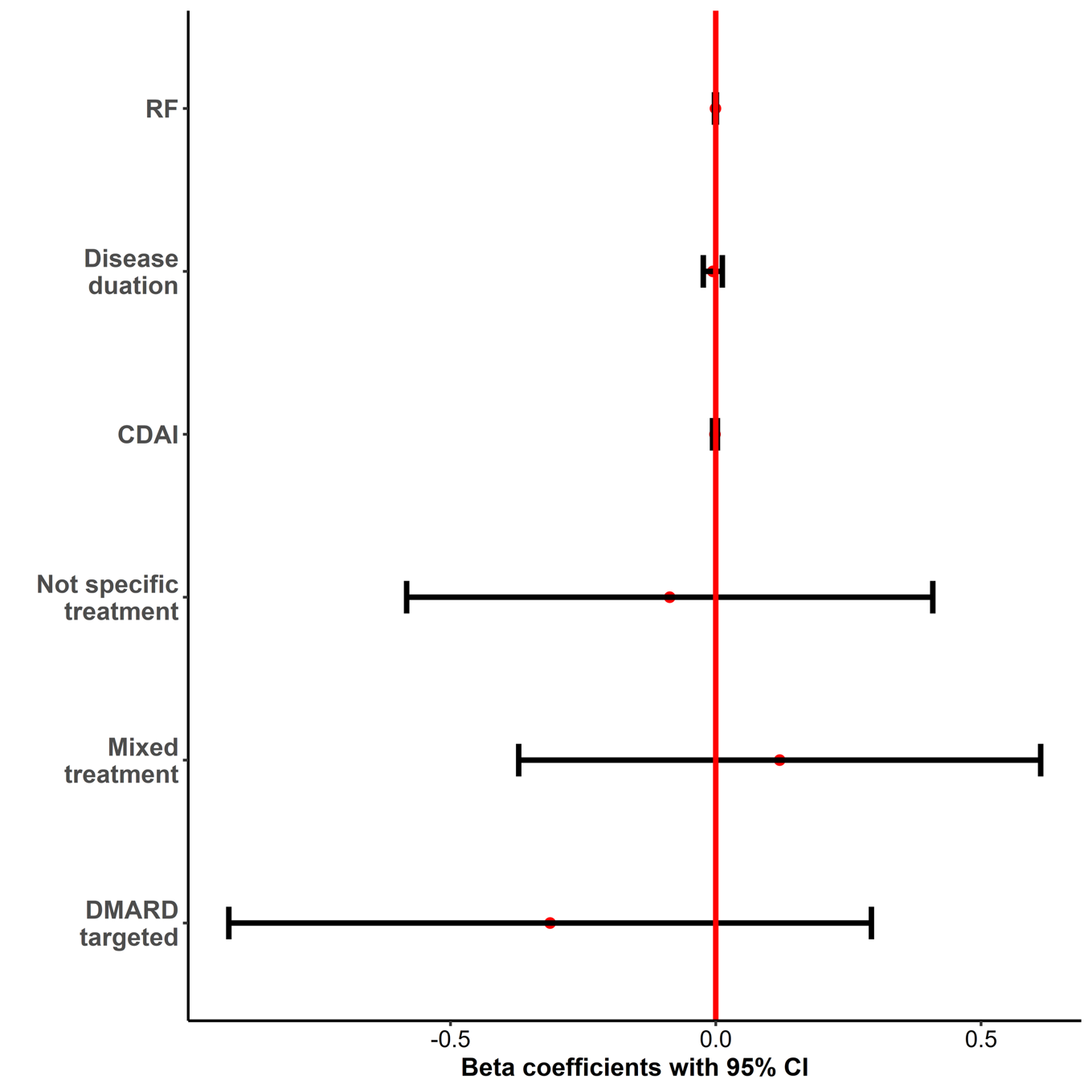


Supplementary figure 8: Impact of different factors on unswitched memory B (_m_B) cell frequency in patients with rheumatoid arthritis (RA) only.


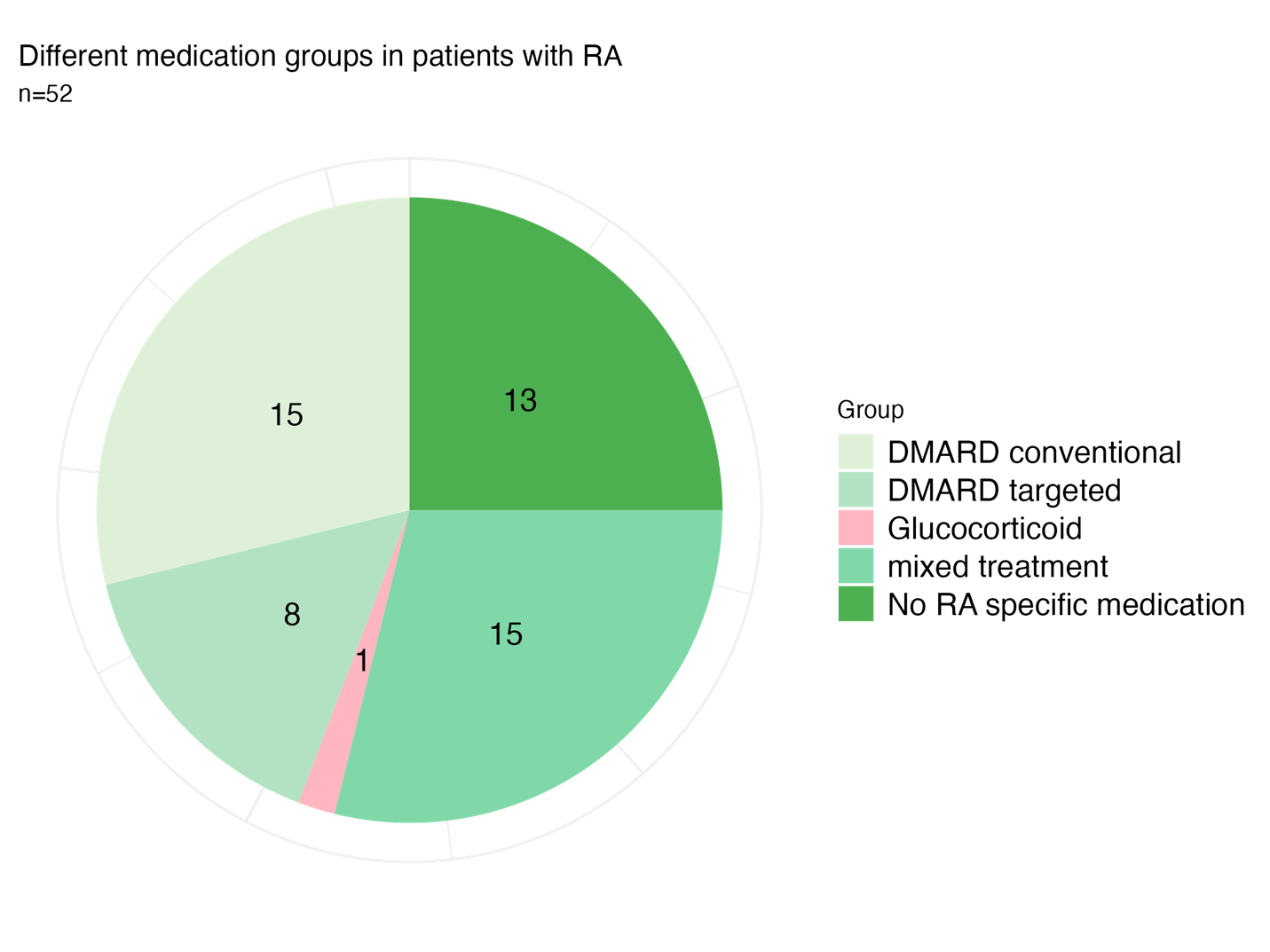


Supplementary figure 9: Different treatment groups. Patients with RA were grouped into five different treatment groups according: conventional DMARDs, biological and targeted synthetics DMARDs, glucocorticoids, mixed treatment and no specific RA medication. N=52. The mean days per dose per group were as follows: DMARD conventional = 6.26 days; DMARD targeted = 14.14 days and Glucocorticoid 1.56 days.
